# Tolerance to the Neuron-Specific Paraneoplastic HuD Antigen

**DOI:** 10.1371/journal.pone.0005739

**Published:** 2009-06-03

**Authors:** Ilana DeLuca, Nathalie E. Blachère, Bianca Santomasso, Robert B. Darnell

**Affiliations:** Howard Hughes Medical Institute and Laboratory of Molecular Neuro-Oncology, The Rockefeller University, New York, New York, United States of America; National Institutes of Health, United States of America

## Abstract

Experiments dating back to the 1940's have led to the hypothesis that the brain is an immunologically privileged site, shielding its antigens from immune recognition. The paraneoplastic Hu syndrome provides a powerful paradigm for addressing this hypothesis; it is believed to develop because small cell lung cancers (SCLC) express the neuron-specific Hu protein. This leads to an Hu-specific tumor immune response that can develop into an autoimmune attack against neurons, presumably when immune privilege in the brain is breached. Interestingly, all SCLC express the onconeural HuD antigen, and clinically useful tumor immune responses can be detected in up to 20% of patients, yet the paraneoplastic neurologic syndrome is extremely rare. We found that HuD-specific CD8+ T cells are normally present in the mouse T cell repertoire, but are not expanded upon immunization, although they can be detected after *in vitro* expansion. In contrast, HuD-specific T cells could be directly activated in HuD null mice, without the need for *in vitro* expansion. Taken together, these results demonstrate robust tolerance to the neuronal HuD antigen *in vivo*, and suggest a re-evaluation of the current concept of immune privilege in the brain.

## Introduction

Immune tolerance to self antigens is believed to protect against autoimmunity, but not in the central nervous system (CNS), which has been widely accepted to be an immune privileged site since Medawar discovered that transplanted allografts were rejected in the body but survived in the CNS [1], [2]. This idea was reinforced when it was recognized that the blood-brain barrier, the absence of lymphatics in the CNS, and the expression of immunosuppressive cytokines and death receptors all serve to protect the brain from potentially harmful inflammatory responses [3], [4]. The mechanisms that might underlie CNS immune privilege have been periodically redefined as new ways of maintaining tolerance have been discovered, and as it has been recognized that CD8+ T cells can infiltrate the brain [5], but the idea remains that the immune system is restricted from seeing brain specific cellular antigens [2]. Although soluble brain antigens readily gain access to the draining cervical lymph nodes where they are taken up by antigen presenting cells, no such pathway has been defined for cellularly-restricted proteins [6], [7]. The immune system, it would seem, is ignorant of antigens sequestered within neurons, providing an explanation of how the brain is protected from potentially damaging autoimmune disease.

The paraneoplastic neurologic diseases (PND) provide an important model of immunity to brain-specific antigens. For example in the Hu paraneoplastic syndrome, patients experience neurologic symptoms that may affect the dorsal root ganglia, limbic system, cerebellum, and brainstem. The neurologic symptoms develop in patients who have small cell lung cancer (SCLC), tumors that express the HuD antigen, and patients generate impressive tumor immunity to this otherwise aggressive malignancy. SCLCs from Hu patients are usually limited to single nodules, and are typically discovered only after presentation of neurologic symptoms [8], [9]. A link between the neurologic symptoms and tumor immunity in the disorder was made by Posner and colleagues, who discovered that these patients harbor high titer antibodies to HuD, a normally neuron-specific RNA binding protein that is also expressed by SCLC cells [10]–[12]. A general model for the disorder has been that the normally neuron-specific expression of the HuD antigen, combined with immune privilege in the brain, accounts for the immunogenicity of the HuD antigen when it is ectopically expressed in SCLC [13]. However, the nature of such immune privilege is poorly understood, and may not explain all aspects of the disorder. For example, even though all SCLC express HuD [11], only some patients make an immune response to the tumor (20%), and very few go on to develop PND [14], as diagnosed clinically and confirmed by the presence of high titer anti-HuD antibodies in the serum and cerebrospinal fluid. While these observations suggest the possibility that there may be mechanisms responsible for inhibiting autoimmunity to HuD, they would also conflict with the idea of immune privilege to neuron-restricted antigens.

The presence of antibodies to Hu in the blood and cerebrospinal fluid are important diagnostic markers for the disorder. However several observations have suggested a T cell mediated component to the PNDs [13]. The Hu proteins are sequestered intracellularly within neurons, activated T cells are present in the cerebrospinal fluid of Hu patients, and the immune response in patients is Th1-skewed, with Th1 CD4 helper T cells and IgG_1_ antibodies present in circulation [15], [16]. All attempts to create mouse models of the disease by immunizing with whole HuD protein have failed to induce neuronal degeneration despite the generation of high titer antibodies to Hu. Moreover, CD8 T cells have been shown to play an important role in the pathogenesis of another form of PND, paraneoplastic cerebellar degeneration [17], [18]. Nonetheless, efforts to identify HuD-specific T cells in SCLC patients with the Hu syndrome have yielded mixed results. Some investigators have reported negative results [19], while others have found inconsistent evidence for such cells specific to affected patients [20]–[22], or the presence of atypical T cells [23]. To examine the nature of the immune response to HuD, we have studied HuD-specific CD8 T cells in the mouse, and found that there is normally robust tolerance to this neuronal protein *in vivo*.

## Materials and Methods

### Mice

Wild type C57BL/6 and Rag1^−/−^ (stock no. 00216) mice were purchased from The Jackson Laboratory. HuD^−/−^ mice [24] (7× backcrossed onto the C57BL/6 background) were obtained from H. Okano.

#### Ethics Statement

All animals were handled in accordance with animal husbandry guidelines established and reviewed by the Rockefeller University Institutional Animal Care and Use Committee (IACUC), which complies with federal and state regulations that concern the use of experimental animals.

### HuD Peptide Screen

C57BL/6 mice were injected in the right footpad with 125 ug of a single HuD peptide emulsified in TiterMax adjuvant. On day 7, CD8+ T cells were isolated from the right popliteal and inguinal lymph nodes by MACS purification (Miltenyi Biotec) and plated in a 20 hour IFNγ ELISPOT assay at 2×10^5^ CD8+ T cells per well. EL4 cells were added as stimulators at 5×10^4^ per well with 10 uM peptide.

### Immunizations

For immunization with recombinant adenovirus, 6–8 week old mice were injected with 100 ul purified adenovirus (10^9^ PFU/mL) i.d. and treated with Pertussis Toxin (Sigma) at days 0 and 2. For immunization with influenza virus, 6–8 week old C57BL/6 mice were injected i.p. with 300 H.A.U. of influenza A/PR/8.

### Preparation of primary murine kidney cells

Kidney cells from adult mice in single cell suspension were plated in 10 cm dishes in D-10 (DMEM supplemented with 10% FBS, nonessential amino acids, sodium pyruvate, glutamine, 22-ME, gentamicin). Cultures were fed by replacing D-10 on days 4 and 7. On day 7, recombinant mouse IFNγ (R & D Systems) was added at 10 U/mL. On day 8, 10 ul recombinant adenovirus at 10^9^ PFU/mL was added the cells were harvested on day 9 for use in an IFNγ Elispot assay.

### Mouse CD8+ T cell stimulation

For *in vitro* stimulation, 2.5–3×10^7^ splenocytes from adenovirus-immunized mice were incubated at 37°C in upright T25 culture flasks (Corning) in R-10 with 0.5 uM peptide for 7 days. For further rounds of restimulation, splenocytes were plated in 24 well plates (2–6×10^5^ splenocytes per well) with peptide-pulsed feeder cells in R-10 with 50 CU/mL recombinant human IL-2 (Chiron). Feeder cells were prepared from spleens of naïve syngeneic mice by pulsing with 0.5 uM peptide for 1 hour at room temperature and irradiating at 3,000 Rads before plating.

### Adoptive transfer

For adoptive transfer experiments, mice received i.v. injections of 7 day *in vitro* stimulated CD8+ T cells (5×10^6^ CD8+/mouse) and DCs pulsed with peptide (2×10^6^ DC/mouse) along with IL-2 (6 injections of 10^5^ CU/mouse i.p. every 12 hours) and Pertussis Toxin (400 ng/mouse i.p. on days 0 and 2). CD8+ cells were isolated from stimulation cultures by negative selection using a CD8+ T cell isolation kit (Miltenyi Biotec). Mature DCs were pulsed with 100 uM peptide in R-10 and incubated at room temperature for 1 hour, shaking every 15 minutes. The cells were then washed twice and resuspended at 10^7^/mL in RPMI 1640 for i.v. injection.

### Mouse DC preparation

Bone marrow-derived DCs were prepared as previously described [25]. On day 7, DCs were matured for 2 days with 125 ng/mL recombinant mouse TNFα (R&D Systems).

### Tetramer staining

Data using surface antibodies (Becton Dickinson) and PE-labeled tetramers (Immunomics iTAg MHC Tetramer, Beckman Coulter) was acquired with a FACScaliber (Becton Dickonson).

### In Vivo CTL assay

Mice received a single i.v. injection of target cells. Targets consisted of two peptide-pulsed populations of CFSE-labeled syngeneic splenocytes in a 1∶1 ratio: CFSE^lo^ syngeneic splenocytes labeled with 0.5 uM CFSE and CFSE^hi^ syngeneic splenocytes labeled with 5 uM CFSE. One population was pulsed with 10 uM HuD p321 peptide and the other with 10 uM βgal p96 peptide. Mice received a single i.v. injection of 200 ul (10^7^ CFSE^lo^ and 10^7^ CFSE^hi^ targets). 7 hours after target injection, the spleens were removed and the amount of CFSE^lo^ versus CFSE^hi^ targets was quantitated by flow cytometry.

### Elispot assay

CD8+ cells were isolated from spleens or lymph nodes of immunized mice using MACS purification (Miltenyi Biotec). T cells were added to IFNγ ELISPOT plates at the indicated concentrations along with stimulators and incubated for 20 hours. Cells were washed out of the ELISPOT plate using a mild detergent followed by incubation with 1 ug/mL biotin-conjugated anti-IFNγ mAb. Wells were developed using the Vectastain Elite Kit according to the manufacturer's instructions (Vector Laboratories). The ELISPOT plate evaluation was performed by an independent evaluation service (Zellnet Consulting) using an automated ELISPOT reader (Carl Zeiss).

### RMA/S Assay

RMA/S stabilization assay was performed as previously described [26].

## Results

### Comprehensive screen to identify potential mouse H-2b HuD epitopes

**Figure 1:**
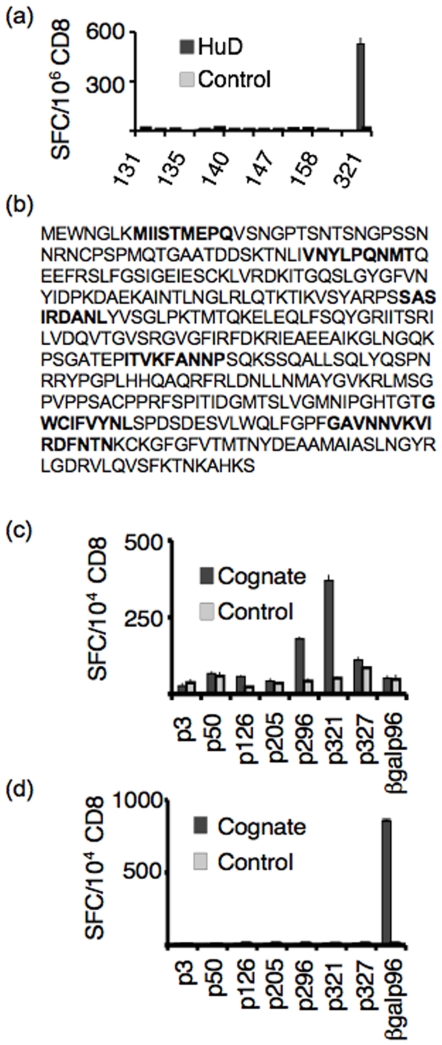
p321 is the immunodominant CD8+ T cell epitope of HuD. (a) A representative peptide screen of 16 HuD peptides. Individual or duplicate C57BL/6 mice were immunized with a single HuD peptide emulsified in TiterMax adjuvant. 7 days later, CD8+ T cells were harvested from draining lymph nodes and plated in an IFNγ ELISPOT assay (2×10^5^/well) with EL4 cells pulsed with 10 uM cognate or irrelevant peptide (5×10^4^/well). The assay was performed in triplicate. Means are plotted and error bars represent standard deviations of the mean. Positive peptides were re-screened in triplicate mice. (b) 7 peptides (in bold) were identified as potential CD8+ epitopes from the HuD protein sequence. (c) C57BL/6 mice were immunized with AdVHuD plus PTx. 13 days after immunization, splenocytes were divided into 8 *in vitro* stimulation cultures and stimulated with each of the 7 HuD peptides or βgal p96. CD8+ T cells were purified from stimulation cultures and plated (10^4^ T cells/well) with cognate or irrelevant peptide-pulsed irradiated EL4 cells (5×10^4^/well) in an IFNγ ELISPOT assay. The assay was performed in triplicate. Means are plotted and error bars represent standard deviations of the mean. Data is representative of three experiments. (d) As a control for *in vitro* priming, C57BL/6 mice were immunized with AdVβgal+PTx and stimulated *in vitro* with each of the 7 potential HuD epitopes or βgal p96 and assayed for IFNγ secretion as in (c).

To determine if HuD-specific CD8 T cells could be detected in the mouse H-2^b^ repertoire, we performed a comprehensive screen of the entire HuD peptide library in C57BL/6 mice. The HuD peptide library consisted of 386 overlapping nonamers, including peptides derived from all known splice variants of the protein. To ensure that all HuD peptide-specific CD8 T cell clones present in the H-2^b^ repertoire could expand, mice were immunized subcutaneously in the footpad with each individual HuD peptide emulsified in TiterMax adjuvant. CD8 T cells isolated from the draining lymph nodes were assessed for their ability to secrete IFNγ in response to cognate peptide-pulsed syngeneic stimulators (Fig 1a). These experiments identified 7 potential HuD CD8 T cell epitopes (Fig 1b).

### Identification of the immunodominant H-2b HuD epitope

We sought to identify immunodominant CD8 T cell epitopes from the 7 candidate HuD peptides. Mice were immunized with replication-deficient recombinant adenovirus expressing full length HuD to allow for *in vivo* processing and presentation of HuD peptides onto MHC I molecules, and splenocytes from immunized animals were stimulated *in vitro* for 7 days with each peptide. Of the 7 potential HuD epitopes, peptides 296 and 321 were able to prime CD8 T cells to secrete IFNγ after adenovirus-HuD immunization (Fig 1c). These CD8 T cell responses were not the result of *in vitro* priming, since HuD peptides failed to elicit IFNγ secretion from mice immunized with a control antigen (adenovirus-β-gal; Fig 1d). Subsequent assays comparing HuD p296-specific and p321-specific CD8 T cells indicated that p296-specific CD8 T cells are low affinity cells, difficult to propagate *in vitro*, whereas p321-specific CD8 T cells can be reproducible and stably maintained in culture (data not shown), and we therefore focused on the latter.

### Characterization of HuDp321-specific CD8 T cells

**Figure 2:**
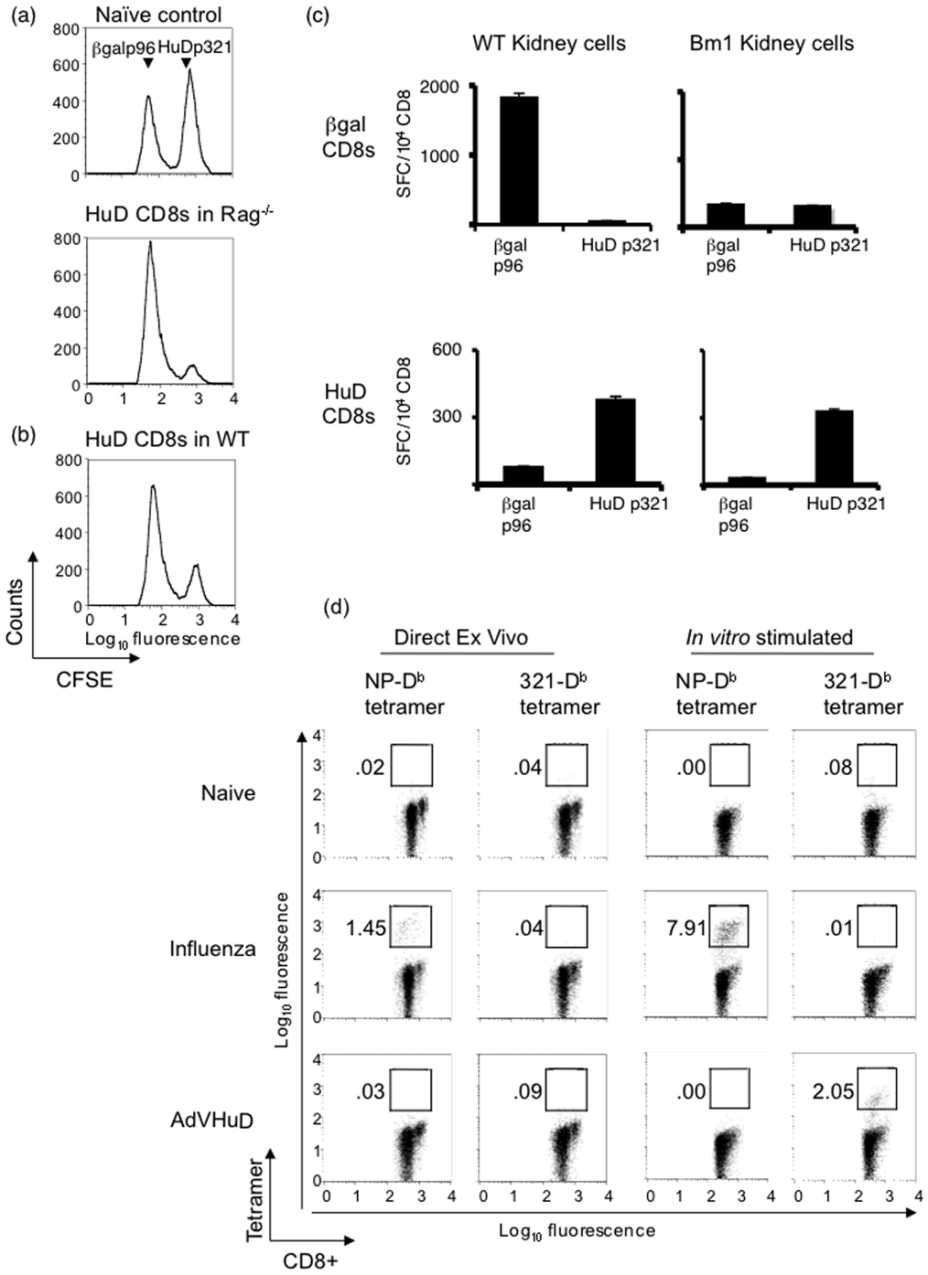
Characterization of HuD p321-specific CD8+ T. (a) 5×10^6^ HuD p321-specific *in vitro* stimulated CD8+ T cells were adoptively transferred into Rag^−/−^ mice (n = 2) with 2×10^6^ C57BL/6 DC pulsed with p321. Mice also received PTx and IL-2. Eight days post transfer, mice were injected with CFSE-labeled syngeneic splenocytes pulsed with HuD p321 (CFSE^hi^) or βgal p96 (CFSE^lo^). A naïve control mouse without transferred CD8+ T cells was injected with CFSE-labeled splenocytes. 6 hours after target injection, splenocytes were analyzed by FACS for *in vivo* target cell lysis. A representative mouse is shown. Data is representative of two experiments. (b) C57BL/6 mice (n = 2) were used as recipients of adoptively transferred HuD p321-specific CD8+ T cells as in (a). A representative mouse is shown. Data is representative of two experiments. (c) Primary kidney cells from C57BL/6 mice (D^b^+/K^b^+) or transgenic Bm1 mice (D^b^+/K^b^−) were irradiated and pulsed with HuD p321 or βgal p96 and used as stimulators in an IFNγ ELIPOST assay (5×10^4^/well) with 3× restimulated HuD p321-specific or βgal p96-specific CD8+ T cells (10^4^/well). The assay was performed in triplicate. Means are plotted and error bars represent standard deviations of the mean. Data is representative of two experiments. (d) C57BL/6 mice were immunized with either AdVHuD or influenza virus or left untreated (2 mice per group). 15 days after immunization, CD8+ T cells were isolated from the spleen and stained directly *ex vivo* with anti-CD8+ antibody and PE-labeled tetramer. A portion of splenocytes from each mouse was stimulated *in vitro* with cognate peptide for 7 days. Naïve mice were stimulated with HuD p321. CD8+ T cells from *in vitro* stimulation cultures were stained with anti-CD8+ antibody and PE-labeled tetramer. Plots are gated on CD8+ T cells. Data is representative of two experiments.

In order to assess whether HuD p321-specific CD8 T cells were able to lyse target cells in an antigen dependent manner, we performed an *in vivo* cytotoxic T lymphocyte (CTL) assay (Fig 2). *In vitro* stimulated HuD p321-specific CD8 T cells were adoptively transferred intravenously into a syngeneic host and assessed for their ability to lyse a population of peptide-pulsed target cells. In order to enhance the effector function of our transferred T cells, we co-injected mature C57BL/6 dendritic cells (DC) pulsed with p321 into hosts. We assessed target lysis in two different strains of mice: wild type C57BL/6 mice and Rag^−/−^ mice. Transferred HuD-specific CD8 T cells were potent cytotoxic effectors in both strains, exhibiting 88% and 74% specific lysis of targets in Rag^−/−^ mice (Fig 2a) and wild type mice (Fig 2b), respectively. Adoptive transfer of HuD p321-specific CD8 T cells together with DC pulsed with irrelevant peptide into Rag^−/−^ mice yielded a lower level of *in vivo* cytotoxicity (20%; data not shown), suggesting that the DC pulsed with p321 served to expand the adoptively transferred population of HuD specific CD8 T cells in recipient mice. These results demonstrate that after activation by immunization and *in vitro* stimulation, HuD p321-specific CD8 T cells are able to function as cytotoxic effectors *in vivo*.

We defined the MHC restriction of HuD p321-specific CD8 T cells in order to design tetramers able to bind their T cell receptors. p321 was predicted to bind D^b^ MHC I on the basis of its amino acid sequence (http://www.syfpeithi.de/). To formally assess the MHC I restriction, we took advantage of the H2^bm1^ mouse strain, which expresses a mutated form of the K^b^ allele. HuD p321-specific CD8 T cells were able to respond to both wild type and H2^bm1^ cells pulsed with cognate peptide (Fig 2c). In contrast, β-galp96-specific CD8 T cells recognized peptide-pulsed wild type cells but not H2^bm1^ cells pulsed with cognate peptide, since the p96 epitope is K^b^-restricted. These results confirm that p321 is presented on D^b^ MHC I.

We were then able to generate a D^b^-p321 tetramer that bound to HuD-specific CD8+ T cells (data not show, and Fig. 2d). We used this tetramer to monitor the expansion of HuD-specific CD8+ T cells after immunization with adenovirus-HuD. For comparison, mice were immunized with influenza virus to track the expansion of NP-specific CD8+ T cells (NP is an influenza epitope that binds D^b^ MHC I). When we measured the frequency of NP-specific CD8+ T cells directly *ex vivo*, we observed an expected frequency of NP-specific T cells (1.5% of the total CD8+ T cell repertoire), and this population was further expanded upon *in vitro* stimulation with NP (Fig 2d). We also found a similar degree of expansion of β-gal-specific CD8+ T cells directly after immunization with adenovirus-β-gal (data not shown). In contrast, after immunization with adenovirus-HuD we saw almost no detectable proliferation of HuD p321-specific CD8+ T cells directly *ex vivo*. Strikingly, however, we were able to expand the population of HuD p321-specific CD8+ T cells upon *in vitro* stimulation to 1.4% of total CD8+ T cells (Fig 2d). The proliferation of HuD-specific T cells depended on the presence of antigen *in vivo*, since naïve splenocytes did not expand after *in vitro* stimulation with p321. Thus, T cells specific for the neuronal antigen HuD do not respond to antigen in a similar manner as T cells specific for a neoantigen; after immunization with adenovirus-HuD, CD8+ T cells are not detectable directly *ex vivo*, but are after *in vitro* expansion, suggesting that their expansion is suppressed *in vivo*.

### Pertussis toxin and in vitro stimulation are required for HuD-specific CD8+ T activation

**Figure 3:**
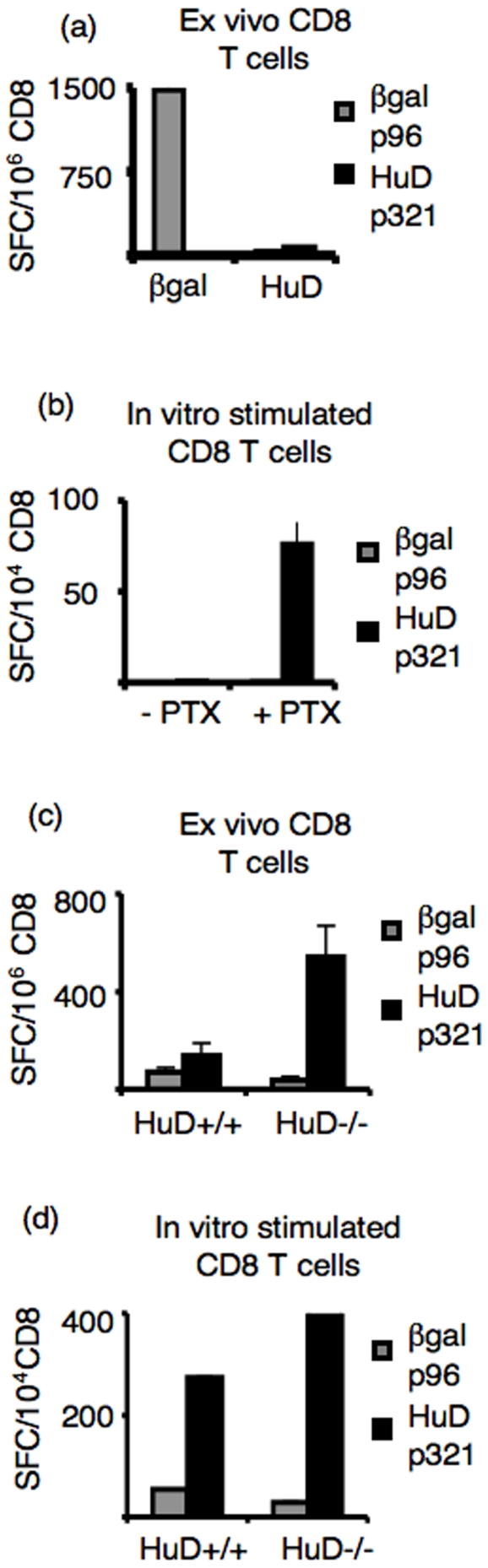
C57BL/6 mice are tolerized to HuD. (a) C57BL/6 mice were immunized with AdVHuD or AdVβgal+PTx (2 mice per group). 13 days later, CD8+ T cells were isolated from the spleen and plated in an IFNγ ELISPOT assay (2×10^5^/well) with EL4 pulsed with 10 uM peptide (5×10^4^/well). The assay was performed in triplicate. Means are plotted and error bars represent standard deviations of the mean. Data is representative of four experiments. (b) C57BL/6 mice were immunized with AdVHuD−/+PTx (2 mice per group). 13 days later, splenocytes were stimulated *in vitro* with 0.5 uM HuD p321. On day 7, CD8+ T cells were plated in an IFNγ ELISPOT assay (10^4^/well) with DC pulsed with 10 uM peptide (7×10^3^/well). The assay was performed in triplicate. Means are plotted and error bars represent standard deviations of the mean Data is representative of two experiments. (c) Individual HuD^+/+^ or HuD^−/−^ mice were immunized with AdVHuD+PTx and used in an IFNγ ELISPOT assay as described in (a). The assay was performed in triplicate. Means are plotted and error bars represent standard deviations of the mean. Data is representative of four experiments. (d) Half of the spleens from mice immunized in (c) were stimulated *in vitro* with HuD p321. After 7 days, CD8+ T cells were isolate from stimulation cultures and plated in an IFNγ ELISPOT (10^4^/well) with peptide pulsed EL4 cells (5×10^4^/well).

To assess the functional status of HuD-specific CD8+ T cells we compared their ability to produce IFNγ after priming *in vivo*. Mice were immunized with adenovirus-HuD or adenovirus-β-gal in combination with pertussis toxin, which was used as an adjuvant to boost the HuD-specific T cell response, and CD8+ T cell IFNγ secretion activation was measured directly *ex vivo*. After immunization with adenovirus-β-gal we saw robust IFNγ secretion by β-gal p96-specific CD8+ T cells. In contrast, we saw no IFNγ secretion by HuD p321-specific CD8+ T cells directly *ex vivo* after immunization with adenovirus-HuD and pertussis toxin (Fig. 3a). When splenocytes from these immunized mice were stimulated *in vitro*, we then detected IFNγ secretion (Fig. 3b), indicating that these cells were able to recognize antigen *in vivo*, but were prevented from becoming effector cytotoxic T cells. Interestingly, no IFNγ secretion was detected after *in vitro* stimulation of splenocytes from mice immunized with adenovirus-HuD in the absence of pertussis toxin. The dependence on both pertussis toxin and *in vitro* stimulation for the activation of HuD-specific CD8+ T cells suggests that mice may be tolerized to the HuD protein since their activation requires both additional adjuvant and expansion *in vitro*.

### Mice are tolerized to HuD

To assay for tolerance to HuD, we immunized HuD-deficient mice [24] (HuD^−/−^) or wild-type littermates (HuD^+/+^) with adenovirus-HuD plus pertussis toxin and looked for IFNγ secretion by HuD p321-specific CD8+ T cells directly *ex vivo*. Whereas HuD^+/+^ mice were unable to generate activated HuD p321-specific cells after immunization, HuD^−/−^ mice generated strong HuD p321-specific CD8+ T cell responses directly *ex vivo* (Fig 3c). To assess whether the lack of an *ex vivo* response in HuD^+/+^ mice was due to ineffective immunization, splenocytes stimulated *in vitro* with p321 were assayed and found to be competent to generate activated HuD p321-specific CD8+ T cells (Fig 3d). The ability to prime this robust *ex vivo* HuD-specific T cell response in mice lacking the HuD antigen, in contrast with the inability of wild-type animals to activate HuD-specific CD8+ T cells in the absence of pertussis toxin plus *in vitro* stimulation, supports the conclusion that wild-type mice are tolerized to the neuronal antigen HuD.

**Figure 4:**
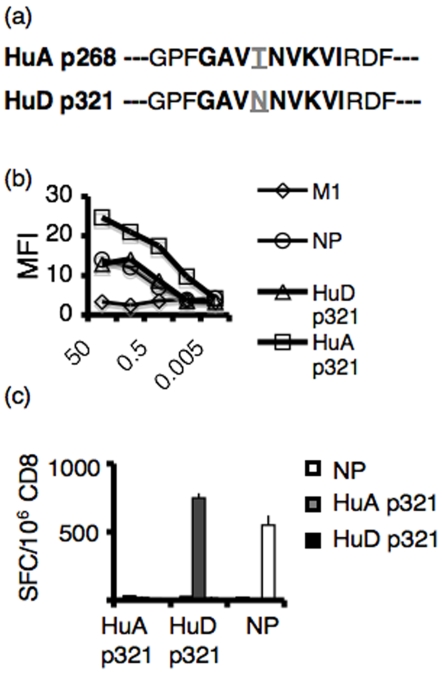
Comparison of HuA p321-specific CD8+ T cells and HuD p321-specific CD8+ T cells. (a) Sequences of HuD p321 and HuA p321 (b) RMA/S cells were incubated with serial dilutions of peptide and stained for D^b^ MHC I. HuD p321 and HuA p321 were assayed. The A2.1 epitope of influenza (M1) was used as a negative control. The D^b^ epitope of influenza (NP) was used as a positive control. (c) C57BL/6 mice were immunized with individual peptides (NP, HuA p321, or HuD p321) in TiterMax adjuvant (2 mice per group). 7 days later, draining lymph node CD8+ T cells were plated in an IFNγ ELISPOT assay (2×10^5^/well) with peptide pulsed EL4 cells (5×10^4^/well). The assay was performed in triplicate. Means are plotted and error bars represent standard deviations of the mean. Data is representative of four experiments.

We compared the CD8+ T cell response to HuD and HuA, a closely related Hu-family member that is expressed ubiquitously throughout the body. In the p321 region of the two proteins, HuA differs from HuD at a single amino acid residue (Fig 4a), although the anchor residues that stabilize the peptide-MHC interaction are conserved between the two peptides, suggesting that they might bind MHC I with similar affinity. We tested the affinities of HuA p321 versus HuD p321 for MHC I using RMA/S cells incubated with serial dilutions of peptide (Fig 4b). HuA p321 had a higher avidity for Db MHC I compared to HuD p321, and HuD p321 bound D^b^ with similar affinity to the control peptide NP. To determine if HuA p321-specific CD8+ T cells are present in the H-2^b^ repertoire, we immunized C57BL/6 mice with peptide emulsified in TiterMax and looked seven days later for IFNγ secretion by CD8+ T cells plated with peptide-pulsed stimulators. Whereas both HuD p321-specific T cells and NP-specific T cells were detectable by IFNγ secretion, no HuA p321-specific T cells could be detected (Fig 4c). These results suggest that T cells specific to the peripheral HuA protein have been deleted from the repertoire or are subject to a more complete and irreversible form of tolerance relative to HuD-specific T cells. Conversely, the ability to detect HuD p321-specific CD8+ T cells confirms that these cells are not deleted by a central tolerance mechanism.

## Discussion

Autoimmune attack against neurons is particularly detrimental, since neurons have limited capacity for regeneration and damage may therefore be permanent. This is clearly the case in the PNDs, where the clinical outcome in these cancer patients is typically determined by the neurologic disease. In the severest forms of the Hu syndrome, patients survive an average of only 7 months from the onset of their illness. Dogma in the past ∼60 years has been that the immune system is ignorant to neuron-specific proteins such as the Hu antigen. However, it has become clear that the HuD antigen, as well as other PND antigens, may be commonly expressed outside of neurons in common cancer types (all small cell lung cancers express HuD, and over half of ovarian cancers express cdr2) [27]. Although only rare subsets of these cancer patients ever develop PND, it would seem that reliance on ignorance of neuronal proteins would pose a potentially great immunologic risk, as neurons induce MHC I molecules during inflammation, at the same time that activated T cells are allowed entry to the CNS across the blood brain barrier [3], [5], [28], [29]. In setting out here to look for HuD-specific T cell responses we have discovered an unexpected resolution to these conflicting points by finding that there is strong tolerance to the HuD protein in normal resting mice. Tolerance to HuD was confirmed by finding that HuD-deficient mice generate functional HuD p321-specific CD8+ T cells directly *ex vivo*, without the need for *in vitro* stimulation with peptide. This tolerance was functionally relevant, as mice examined after receiving HuD immunizations or at various times after HuD CD8+ T cell adoptive transfer (9 days, 18 days, one month) did not develop evidence of neurologic dysfunction or abnormalities evident by immunohistochemical studies of brain sections.

The presence of HuD p321-specific CD8+ T cells in the H-2^b^ repertoire indicates that these cells are either not subject to central tolerance induction in the thymus, or exist as a result of incomplete central tolerance. Extensive examination of HuD expression in the adult mouse has failed to find protein in the thymus or any other tissue outside of the nervous system [30], and HuD does not appear to be regulated by the AIRE protein [31]. In addition, we immunized AIRE KO mice with adenovirus-HuD and saw no HuD p321 specific T cell directly ex vivo (data not shown). In contrast, when we compared the CD8+ T cell response to HuD p321 versus its homologous epitope in HuA, we found that HuA p321-specific CD8+ T cells were not detected after TiterMax immunization, suggesting that they were centrally deleted. Given HuA's ubiquitous expression pattern, central tolerance would be important to prevent the development of widespread peripheral autoimmunity, which is not observed in HuD patients. In contrast, HuD p321-specific CD8+ T cells were present in the repertoire; moreover, the IFNγ response of HuD p321-specific T cells was comparable in magnitude to that elicited from neoantigen-specific T cells after Titermax-peptide immunization. While this experiment was done with a high concentration of peptide (1×10^−5^ M), which could have obscured low affinity T cell responses, we found that the affinity of restimulated HuD and βgal T cells were comparable (in peptide ELISPOT titration assays, both recognized targets pulsed with dilutions of peptide down to 1×10^−9^ M; data not shown). Taken together, our data point to the possibility that mice normally have a mechanism for maintaining active tolerance to a neuron-specific protein.

The maintenance of tolerance, either by regulatory T cells or through induction of anergy of CD8+ T cells in the periphery, requires a means for antigen to be captured and presented to antigen presenting cells (APCs), which themselves make no detectable HuD mRNA (unpublished data). Given the restricted expression of HuD to neurons, which do not turnover in the steady state, the means of sustaining peripheral tolerance to HuD would seem dependent on either ectopic expression of the protein by a non-neuronal cell type, or cross presentation of HuD by an APC. Examination of HuD expression in adult mouse tissue has confirmed its restriction to neurons [30] (Darnell et al., unpublished data), although it is difficult to exclude the possibility that an undetectable amount of protein may have escaped detection in these negative results. Recently, ectopic expression of peripheral-tissue antigens by lymph node stromal cells was reported [32]. In this transgenic mouse model, a cytosolic form of ovalbumin (OVA) under the control of a tissue-specific promoter was shown to be endogenously processed and presented onto MHC I molecules by a stromal cell population in the lymph node cortex. Endogenous presentation of OVA in the lymph node was an effective means of inducing tolerance in naïve OT-I CD8+ T cells. Circulating HuD-specific CD8+ T cells may be subject to a similar form of regulation if ectopic expression of HuD by lymph node cells resulted in presentation of HuD p321 peptide complexes, although we have no expression data to support such a speculation.

Soluble CNS antigens may drain to cervical lymph nodes, and proteins in the CNS can readily access lymphoid tissues via the CSF and cervical lymphatics for processing and presentation to the immune system [2], [29], [33], [34]. However, it is not clear that this pathway is relevant to an intracellular neuronal protein like HuD. Another possibility is that phagocytosis of HuD-expressing neurons, or fragments of neurons such as remodeling synapses, could provide a means for antigen entry into an APC. While whole neurons may not be a likely form of antigen for APCs given their limited ability to turn-over, APCs may be able to phagocytose membrane-bound neuronal blebs for cross presentation and subsequent tolerance induction, in a manner analogous to their general ability to cross-present antigen from apoptotic cells [18], [35]. For example, outer segments of photoreceptors, normally phagocytosed by retinal pigment epithelium, may instead be phagocytosed by microglia following transplantation into the brain [36], [37].

We have shown that a protein confined in expression to neurons is not ignored by the immune system but undergoes tolerance induction in order to prevent against autoimmunity directed at the nervous system. The maintenance of tolerance to HuD is apparently quite effective, given that all SCLC express the HuD antigen but only a very small (0.01%) population of patients succumbs to neuronal degeneration. Tolerance to HuD suggests revisiting the model of the brain as an immune privileged organ. Rather, we suggest the opposite possibility, that there exists a natural mechanism that acts in the steady state, similar to what occurs with peripheral tissue antigens [38], but that is perhaps even more profound, to tolerize T cells to brain antigens as a default, and for the same end purpose—to keep the organ from autoimmune attack. In this context, Medawar's data and follow-up studies may be explained by immunologic tolerance to brain antigens, even newly transplanted antigens, rather than by immunologic ignorance as originally suggested.
